# A Community Jury on initiating weight management conversations in primary care

**DOI:** 10.1111/hex.13286

**Published:** 2021-06-21

**Authors:** Rebecca J. Beeken, Anna M. Scott, Rebecca Sims, Gina Cleo, Helen Clifford, Paul Glasziou, Rae Thomas

**Affiliations:** ^1^ Leeds Institute of Health Sciences University of Leeds Leeds UK; ^2^ Centre for Research in Evidence‐Based Practice Bond University Gold Coast Qld Australia; ^3^ Gold Coast Hospital and Health Service Public Health Unit Gold Coast Qld Australia

**Keywords:** community jury, obesity, primary care, weight management

## Abstract

**Background:**

Current guidelines recommend that patients attending general practice should be screened for excess weight, and provided with weight management advice.

**Objective:**

This study sought to elicit the views of people with overweight and obesity about the role of GPs in initiating conversations about weight management.

**Methods:**

Participants with a body mass index ≥25 were recruited from a region in Australia to take part in a Community Jury. Over 2 days, participants (n = 11) deliberated on two interconnected questions: ‘Should GPs initiate discussions about weight management?’ And ‘if so, when: (a) opportunistically, (b) in the context of disease prevention, (c) in the context of disease management or (d) other?’ The jury deliberations were analysed qualitatively to elicit their views and recommendations.

**Results:**

The jury concluded GPs should be discussing weight management, but within the broader context of general health. The jury were divided about the utility of screening. Jurors felt GPs should initiate the conversation if directly relevant for disease prevention or management, otherwise GPs should provide opportunities for patients to consent to the issue being raised.

**Conclusion:**

The jury's verdict suggests informed people affected by overweight and obesity believe GPs should discuss weight management with their patients. GPs should feel reassured that discussions are likely to be welcomed by patients, particularly if embedded within a more holistic focus on person‐centred care.

**Public contribution:**

Members of the public took part in the conduct of this study as jurors, but were not involved in the design, analysis or write‐up.

## 1 INTRODUCTION

Obesity is a risk factor for numerous diseases, including type 2 diabetes, cardiovascular disease and some cancers.^1^, ^2^ In Australia, the prevalence of overweight and obesity has been increasing; 63% of adults were classed as overweight or obese in 2014‐15, up from 57% in 1995.^3^ The Australian National Health and Medical Research Council (NHMRC) clinical practice guidelines for the management of overweight and obesity identify primary care as playing an important role in weight management,^4^ and there are increasing calls for primary care to screen for overweight and obesity.^5^

However, initiating weight management conversations in primary care is a controversial issue for general practitioners (GPs). Despite current guidelines, previous studies suggest few patients attending general practice in Australia are weighed, and few at‐risk patients are given advice or referred to other services.^6^ Identified barriers include insufficient time, concerns about upsetting patients, lack of training/confidence in this clinical area and perceptions that effective interventions are lacking.^7^, ^8^, ^9^ A recent systematic review and thematic synthesis of 29 qualitative studies exploring GPs' and nurses' perspectives on discussing weight with patients with overweight and obesity in primary care highlighted that discussing weight is not perceived to be a priority for GPs.^10^

GPs' perceptions that effective weight management options are lacking, are not entirely unsupported. Although there is some evidence that GP referrals to programmes outside of the primary care setting can be effective, the effects are generally modest. A systematic review of 15 RCTs of behavioural interventions in primary care indicated a mean weight loss of 1.36 kg (−2.10‐−0.63, *P* < .0001) at 12 months and −1.23 kg (−2.28‐−0.18, *P* = .002) at 24 months.^11^ More recently, a UK‐based trial of an opportunistic primary care referral to a behavioural weight‐management programme supported a similar weight loss difference of 1.43 kg (95% CI 0.89‐1.97) over 12 months.^12^ While these effects are not negligible at the population level, individual patients may be disappointed.

Exploring the views of those directly affected by overweight and obesity provides GPs and policymakers with an important perspective on this issue. Data from the UK‐based trial of an opportunistic primary care referral suggested <1% of patients felt it was inappropriate.^12^ Furthermore, cross‐sectional data suggest that receiving advice from a GP may be an important motivator for patients; people who report receiving advice from a health‐care professional to lose weight are more likely to be trying to lose weight than people who do not recall receiving advice.^13^ Two qualitative studies conducted in the United States and Canada also suggest patients want and expect their GP to discuss weight management, but do perceive there to be limitations within current models of care.^14^, ^15^ More research is needed to gain a clearer understanding of patients' views around GPs initiating these discussions, and whether patients perceive it to be appropriate in all circumstances.

Community Juries (CJs) are a deliberative democracy technique that provides a group of community members affected by an issue with expert information and an opportunity to question the experts. The jury then deliberate, to form a ‘verdict’ on the issue, which can influence health policy decision making.^16^ In contrast to other qualitative approaches, a CJ therefore asks those affected by the issue to reflect on more than just their own experiences. The expert information places the issue within a wider context, and participants are asked to consider the issue with the community interest in mind.^17^ Obtaining the views of individuals both affected by the issue, and informed of the context and implications of different approaches via the CJ method, may be more likely to influence GPs and policymakers than views obtained via interviews or focus groups alone.

The aim of this CJ was therefore to elicit the views of people with overweight and obesity about the role of GPs in initiating conversations about weight management. CJ participants deliberated on two questions: ‘Should GPs initiate discussions about weight management?’ And ‘if so, when: (a) opportunistically, (b) in the context of disease prevention, (c) in the context of disease management or (d) other?’

## 2 METHODS

### 2.1 Participants & recruitment

Recommendations for the composition of CJs suggest a sample size between 12 and 25 is appropriate.^18^ We were therefore aiming to recruit 15 participants to allow for some dropout and ensure there were sufficient people to encourage a wide ranging, but manageable, discussion, where all voices would be heard.^19^ We were seeking to obtain the views of ‘consumers’ (the affected public),^17^ as these would be most relevant to GPs considering this issue. Therefore, we recruited participants over 18 years with a body mass index ≥25 (calculated from self‐reported height and weight). We excluded anyone unable to provide informed consent due to mental incapacity, or unable to speak or understand English.

We recruited participants from a region in Australia through Taverner Research using a random‐generated landline and location known mobile sample drawn from SamplePages. This sample frame had the potential to cover 80% or more of the population in the region. Compared with an online panel or market research database, recruits were therefore less likely to have been exposed to research and people without internet could be included. We requested roughly equal numbers of men and women, and where possible, a range of education levels and ages.

Using the random generated and location known telephone numbers, Taverner Research contacted potential participants and, without coercion or pressure, asked respondents whether they would be willing to receive more information about the study. Interested participants were checked by Taverner Research for eligibility, and given further details about the study, including an explanatory statement containing details about the nature and purpose of the CJ alongside contact details for the research team in case they had any queries, and a consent form. If potential participants agreed to take part after reading this sheet, and having had the opportunity to contact the research team with any queries, then they were asked to sign a consent form. Participants were asked to either send the consent form in advance, or bring a hardcopy on the day of jury. Written consent was obtained from all participants before commencing the CJ.

CJ participants received two $100 gift cards as reimbursement for their time. Ethics approval was provided by the University's Human Research Ethics Committee (#16213).

### 2.2 Procedure

The CJ was conducted over a weekend (10‐11 March 2018) (see Table 1 for schedule). All sessions except for the final deliberation were facilitated by an experienced facilitator and researcher, who had conducted work in the field of obesity/weight management. The facilitator ensured equal participation, recorded questions and noted participant concerns. Two observers took notes on participant comments, affect and participation, except during the final confidential deliberation. To not lead or bias the jurors towards a specific recommendation, only jury group members were present during private deliberations.

**TABLE 1:** Community Jury schedule

9.00‐9.30	Overview of Community Jury
9.30‐10.00	What is obesity, what is the prevalence and what are the consequences
10.00‐10.30	Questions
10.30‐11 AM	MORNING TEA
11.00‐11.30	What options are available for weight management in the local region
11.30‐12.00	Questions
12.00‐12.30	LUNCH
12.30‐1.00	Reasons against GPs initiating conversations about weight management
1.00‐1.30	Questions
1.30‐2.00	Reasons for GPs initiating conversations about weight management
2.00‐2.30	Questions
Flexible timing in response to Juror needs	Jury deliberations stage 1
	AFTERNOON TEA
	Questions and close
9.00‐9.30	Reconnect and debrief
9.30‐10.30	Further questions and deliberations
Flexible timing in response to Juror needs	MORNING TEA
	Deliberations until consensus or impasse
	LUNCH
	Deliver verdict
	Debrief, process discussion and close

On Day 1, following written consent, participants completed a brief survey to assess their comprehension of the topic and attitudes prior to receiving information. Four experts with clinical, research and public health expertise, each presented 20‐minute voice over PowerPoint presentations followed by a telephone question–and‐answer session with the jurors. As background, the first expert provided a scientific overview of obesity; the second presented on the available resources and services for weight management in the local region. The third and fourth experts presented opposing views on whether GPs should initiate conversations with patients about weight management. Participants were provided with hand‐outs of the presentations and the experts’ biographies. Participants commenced facilitated discussions after the presentations and broke for the day. On Day 2, participants shared overnight reflections and, where needed, re‐questioned the experts by telephone. Participants then deliberated in private until a consensus or impasse was reached and presented their decisions on the two questions to the facilitator and other researchers. Post‐surveys were administered to participants prior to CJ completion.

### 2.3 Measures

It is important to ascertain that CJ participants made an ‘informed decision’ when providing their recommendations. This confirms the CJ has made their recommendations with the necessary knowledge and understanding of the issue, without which their decisions could be undermined.^20^ Agreed definitions of informed choice suggest this requires adequate comprehension of the topic and consistency between personal attitudes towards the topic and decisions.^21^

Ten comprehension questions relating to information provided during the expert presentations were developed for this study to assess jurors’ comprehension. In line with a previous CJ, adequate comprehension for an informed decision was defined a priori as 50% correct.^22^

Drawing from previous research, attitudes towards GPs initiating a conversation about weight management were measured at both a personal and general level. Participants were asked ‘For me, a GP initiating a conversation about managing my weight would be…’ and ‘In general, GPs initiating conversations about weight management with their patients is…’ and responded to five items on a 7‐point scale with higher numbers suggesting attitudes that are more positive.^21^, ^22^, ^23^ Consistent with previous CJ research, a positive attitude was defined as scores ≥28/35.^22^

To capture jurors' decisions, participants were asked whether they would want GPs to initiate a conversation with them and whether they thought GPs should initiate conversations in general using a 7‐point scale ranging from 1 (definitely not) to 7 (definitely would/should). Responses of 5‐7 were classified as positive and scores of 1‐4 as negative. As per other CJ studies, to explore the time and information provision required to achieve consistent responses, we asked participants these questions on nine occasions.^24^

An informed decision was defined as adequate comprehension (>50%) and a consistency between attitudes and decisions.^21^, ^22^

### 2.4 Analyses

CJ proceedings were audiotaped and transcribed. Reasons for the jury recommendation were analysed by two researchers using thematic analysis.^25^ Changes to participant survey responses pre‐ to post‐CJ were assessed using Wilcoxon signed‐rank tests. All quantitative data were analysed in SPSS Statistics 23.

## 3 RESULTS

Of the 13 participants recruited, 11 (5 males and 6 females) attended the CJ weekend. No explanations were provided for non‐attendance. Mean age of attendees was 47 years (SD 20); median BMI was 29.1 (IQR 26.6‐31.6). All jurors had completed high school and a majority had some post‐high school education (9/11; See Table 2).

**TABLE 2:** Participant demographics (N = 11)

Male n/Female n	5/6
Age mean (sd)	47 (20.1)
BMI median (IQR)	29.07 (26.56‐31.62)
Previous discussion with GP about weight n	
No	2
Yes	
Out of the blue/unprompted	0
Unprompted and health risk	2
Unprompted and health risk related to weight	1
I asked about it	6
Education n	
Finished high school	2
Some post high school or TAFE	4
University or TAFE graduate	5

### 3.1 Community Jury decision, GP initiating conversation on weight management

The jury found the original first question problematic and opted to change it to ‘Should GPs discuss lifestyle, health, and weight management, with their patients?’ All 11 jury members voted yes to the amended question.

In what contexts should GPs discuss weight management? (a) Patient‐initiated (e.g. when a patient brings the topic up for discussion or if the patient has completed a questionnaire that asks if they would like to discuss specific issues with their GP, and lifestyle, weight management or health is one of these topics), (b) In the context of prevention and management of a health condition, and / or (c) Screening (discussed with everyone who comes in, regardless of context/reason for visit).

While all jury members agreed with options (a) and (b), option (c) was more divisive, with five members agreeing and six disagreeing that GPs should screen all patients for overweight/obesity.

### 3.2 Jury's rationale for changing the question

The jury felt it was important that weight management be considered as just one aspect of overall health as part of a holistic approach to care.

‘you don’t have to bring it up just as a problem with weight. You can say, ‘How are you going? How’s everything? What have you been doing lately? How do you feel?’ (2F)

It was perceived that raising the issue in a more general way could avoid negative responses from patients and reduce perceptions of stigmatization.

‘how the GP should say it, so that it’s received in the best possible way. So, that’s why we’re changing the question to lifestyle, yes? So, it’s not as focused on weight, but it’s also looking at all of the factors that are contributing to the overweight or obese situation? It’s all‐encompassing. It’s not selecting certain groups of people, or pointing the finger, ‘You’ve done this wrong, and now you’re fat.’ (13F)

### 3.3 Jury's rationale for verdict

Jurors unanimously agreed a GP was the most appropriate person to discuss weight management with an individual as they could be objective.

‘the doctors have the opportunity that other people don’t. It’s impersonal. They don’t have that emotional connection. I think they’re the best person to start the conversation. If they don’t start, and the family doesn’t do anything, who is going to do it?’ (2F)

The GP was described as someone a patient could trust:

‘if it’s not a doctor, then who? Because, family, they’re just going to irritate people. Friends, they’re just going to try to butter it up. But if it’s not someone that they trust, and everyone trusts their doctor, to a certain degree. They trust they’re educated, they trust they’ve seen it all.’ (11F)

Jurors were also aware of the wider impact of obesity on the community.

‘The implications of the resources in the community that are spent on disease management, to me, warrants this being taken very seriously’ (3F)

The jury recognized that weight management is difficult.

‘there is no definitive treatment for weight. There is nothing that you can say, ‘Well, you go on this drug, or you go to this’ (8M)

Jurors unanimously agreed it would be appropriate if the patient had initiated the discussion and that patients should have some degree of control, whether through initiating the conversation themselves, or providing consent, for example through a pre‐appointment questionnaire.

‘But it really comes down to the consent of the patient. That’s the thing that I feel most strongly about. Just like xxx said, however we do it, whether it’s electronically or paperwork, that something is given to the patient so that they feel like they’re more in control of the discussion that’s going to occur, and it’s not an awkward thing or a discomfort.’ (13F)

Although all participants emphasized consent, they also agreed that there should be circumstances when the GP could raise the issue without the patient initiating this discussion, that is in the context of disease prevention or management.

‘But even if they’re unwilling to discuss it, but even if it doesn’t come up, but they’re getting a blood test…the doctor has that duty of care. So, it’s that weighing up of opportunity, desire and need.’ (12M)

Nevertheless, jurors felt that discussing weight with an unmotivated or uncomfortable patient would be unlikely to help and could cause harm.

‘GPs initiating the conversation on unwilling people, you might be shooting them in the foot, preventing them, causing more issues’ (12M)

In terms of screening, the jury did not reach a unanimous decision. There was a lack of clarity about the appropriate timing of any potential weight screening, or evidence for this approach.

‘the screening is just a mechanism, and how it’s applied and when, is not answered’ (7M)

‘That’s fine and dandy to screen every human being, everybody who walks through their door, but we don’t. We don’t know what works.’ (12M)

Around half felt this would be appropriate as they believed GPs should be able to discuss weight in all contexts.

‘I just think that when the opportunity arises on anything, that they should be able to confront you. They're your GP.’ (5F)

Those in support of screening felt that screening could be normalized, or made routine, in line with other regular checks, and that weighing everyone would avoid discrimination.

‘Don’t a lot of doctors take your blood pressure every time you walk into there anyway, so why can’t they use the scales? (11F)

Participants for screening also suggested this could provide an opportunity to discuss the issue with patients who may not otherwise visit their GP, and was perceived as potentially helpful to the individual.

‘I still reckon that should be put in the place. The reason being, a lot of people don’t go to doctors.’ (10M)

If you’re going to the doctors’ twice a year, or once a year, and over five or ten years, you can see a progressive up or down, or you’re staying the same, I think personally, that’s a good thing to go back and look at.’ (11F)

Those not supportive of screening felt this approach would conflict with giving patient's control over these discussions.

‘I think it’s in direct contradiction with the patient‐initiation. So, I think it’s a moot point’ (13F)

Participants against screening also raised concerns about the reliability of BMI as a screening tool:

‘However high you are, I’m stocky, another guy is skinny. There’s a difference straight away. It doesn’t mean anything, but I’m not going to go along and have someone take my picture and write me a letter saying I’m fat. It’s hard to judge how fat you might be’ (7M)

### 3.4 Comprehension, attitudes and individual decisions

There were no changes pre‐ to post‐CJ in participants’ comprehension, attitudes or interest in GPs initiating conversations about weight management with either themselves or other patients (Table 3). On average, comprehension scores were high at both pre‐ and post‐CJ (median = 8/10, IQR = 2; and median = 8, IQR = 1, respectively). Attitudes towards GPs initiating conversations with either themselves or with other patients were rated as somewhat negative (<28/35) pre‐CJ (for themselves median = 27, IQR = 6, and with other patients median = 27, IQR = 12) but more positively (≥28/35) post‐CJ (for themselves median = 33, IQR = 10, and with other patients median = 31, IQR = 10). CJ participants wanted GPs to initiate a conversation *with them* about managing their weight both pre‐ (median = 7/7, IQR = 2) and post‐CJ (median = 7, IQR = 2, *P* = .78) and this was similar for GPs initiating conversations with patients in general (median = 6/7, IQR = 2, and median = 7, IQR = 2, respectively, *P* = .52).

**TABLE 3:** Knowledge and attitude scores pre‐ and post‐Community Jury (N = 11)

	Pre‐assessment			Post‐assessment			Wilcoxon *P*‐value
	Median	Q1	Q3	Median	Q1	Q3	
Knowledge total (/10)	8	7	9	8	8	9	.20
Attitudes							
(discussion w/individual) (/35)	27	26	32	33	25	35	.33
Attitudes							
(discussion w/other patients) (/35)	27	23	35	31	25	35	.27

### 3.5 Informed decision

Eight participants made an ‘informed decision’ about GPs initiating a conversation with them personally, one participant had a low score on the attitude scale suggesting a negative attitude but positive interest in a GP initiating a weight management conversation, and two participants had borderline scores. For whether GPs should initiate a conversation with patients in general about weight management, nine participants were deemed to have made an ‘informed decision’, one borderline and one uninformed.

## 4 DISCUSSION

A CJ of individuals affected by overweight and obesity concluded unanimously that GPs should be discussing weight management in primary care, but within the broader context of general health. To our knowledge, this is the first time a CJ has explored this issue. In contrast to other qualitative approaches, participants were provided with information from experts on the pros and cons surrounding this issue, and encouraged to deliberate. At the end of the process, the majority had made an informed decision. Policymakers should feel reassured that this jury were supportive of current guidelines for primary care to play a role in weight management, and GPs less reluctant to act on this. Previous research suggests physicians are concerned about offending their patients if they discuss weight management ^7^, ^26^ and do not see it as priority.^10^ However, this study suggests discussions may be welcomed if the focus is on health, and patients are able to decline such conversations. Moreover, many jurors felt it would be the doctor's duty to have those conversations. This positive attitude towards GPs involvement in weight management aligns with other studies ^14^, ^15^, ^27^, ^28^ and a recent trial of a brief intervention for obesity in primary care in the UK.^12^ In this trial, patients not only welcomed a weight management referral from their GP, but also lost weight.^12^ Together, these results suggest GPs should feel confident that having weight management conversations with their patients has value.

The jury were divided on whether patients should be routinely screened for overweight/obesity; some perceived this to be non‐discriminatory and helpful, whereas others felt it took control away from patients and were sceptical about the usefulness of BMI as a screening tool. Current guidelines for screening for overweight/obesity suggest all patients should be assessed, but only after the patient has given permission.^4^ This is in line with the consistent position of this jury around the importance of patient consent. GPs seeking to implement screening could build a consent process into brief pre‐appointment questionnaires, enabling patients to indicate readiness for a discussion. This would support a person‐centred approach that considers obesity management over time as opposed to within a single appointment.^29^

Framing any questionnaire or discussion around well‐being and general health was perceived by the jury to be more acceptable than a sole focus on weight. All jury members were positive about weight management being raised in the context of disease, but discussed this in relation to clear indicators (such as high blood pressure). This is in contrast to some research, which suggests attempts to link weight management discussions to health may be more likely to cause resistance.^26^ However, the jury was more divided about BMI alone as a risk factor and screening tool, and emphasized the importance of considering all aspects of an individual's health and well‐being. On the other hand, routine screening for all patients was discussed as a way of avoiding reliance on a GP’s visual assessment alone, which can be inaccurate,^30^ and could cause patients to feel stigmatized.

Another common barrier for GPs discussing weight is the perception that interventions are ineffective, particularly over the longer term.^7^ However, while some members of the jury acknowledged that weight management is difficult and outcomes variable, no jury members voiced that they felt this should be a deterrent. Some members of the jury highlighted that GPs are perceived as both trustworthy and objective. Patients may be open to discussions about weight management options presented alongside the available evidence. Additionally, highlighting the independent value of increasing physical activity for health,^31^ for example, may be well‐received. In this context, it may be important to incorporate measurements of success beyond weight loss and to assess the impact of any changes on overall well‐being.^32^

It is important to note that this jury chose to change the question provided to them. Previous public deliberation studies have permitted participating publics to frame the questions,^17^ but it is not a common outcome. Involving members of the public with lived experience in the design of future CJ research could ensure the development of questions that a jury feels are appropriate. In the context of the current study, the change could reflect the complexity of the issue, and sensitivity around the language used when discussing weight management.^33^ The jury felt that asking about weight management alone was insufficient, and potentially stigmatizing. As the jury consisted of people affected by overweight and obesity, it was crucial that the question be posed in a way that was perceived by them as non‐discriminatory. If, as researchers, we had not been open to this change, members of the jury may have disengaged. However, it does highlight that a response to the initial question may have been less unanimous, and both policymakers and GPs should bear these nuances in mind. Training may help GPs to navigate some of the more subtle, and delicate aspects of discussing weight management appropriately.^34^

Community juries are not intended to be representative of the wider population but we sought to include a balance of men and women, and a variety of ages and levels of education. However, a limitation of this study is that the majority of our sample were educated to a post‐high school level and those with lower levels of education were under‐represented. Due to low recruitment and 2 non‐attenders on the day, our sample was below the recommended composition of 12‐25. It was still within the range reported within a scoping review of public deliberation, which identified 20 community juries, most of which were 1‐2 day events involving a single jury of 9‐16 people.^17^ However, the low recruitment may suggest a lack of interest from individuals affected by overweight and obesity in this issue or that our approach was not appropriate. In line with the recruitment processes for most CJs,^17^ we used an external recruitment company. This company did not hold information on our eligibility criteria, including BMI, and so we do not have data on eligibility rates nor on the number eligible that were approached but declined. The median BMI for our sample was within the overweight category, and we may not have captured the views of those affected by higher levels of excess weight. People with severe obesity are more likely to experience stigma, including in health care,^35^ and so may have been less willing to discuss their experiences. Future research should seek to engage those with higher BMIs, to explore whether they would make similar decisions. All participants in this jury also had high levels of knowledge about overweight and obesity at baseline. Their views provide insight into the informed attitudes of people affected by overweight and obesity. However, other, less‐engaged groups may reach different conclusions. Similarly, the views expressed were based on engagement with primary care in Australia, and it will be important to repeat this process in countries where experiences of primary care may differ. This jury also focussed on GPs discussing weight management within primary care and did not explore views on other health‐care professionals engaging patients in these discussions.

The present study suggests that informed people affected by overweight and obesity in Australia believe GPs should discuss weight management with their patients. The views of this jury are in line with the NHMRC clinical practice guidelines for the management of overweight and obesity in Australia, which recommend GPs Ask, Assess, Advise, Agree and Assist.^4^ GPs should feel reassured that this approach is likely to be welcomed by patients, particularly if embedded within a more holistic focus on person‐centred care.

## CONFLICT OF INTEREST

None declared.

## AUTHOR CONTRIBUTIONS

RB, AS, RT and PG designed the study. RB, AS, RT, GC, RS and HC contributed to the running of the study. RB, AS and RT analysed and interpreted the data. RB wrote the manuscript. All authors provided critical comment, edited the manuscript and approved its final version.

## Data Availability

The data that support the findings of this study are available from the corresponding author upon reasonable request.
